# Cognition comes of age: comments on the new FDA draft guidance for early Alzheimer’s disease

**DOI:** 10.1186/s13195-018-0386-7

**Published:** 2018-06-29

**Authors:** John E. Harrison

**Affiliations:** 1 Metis Cognition Ltd., Kilmington Common, Wiltshire, BA12 6QY UK; 2 Alzheimer Center, Amsterdam, The Netherlands; 3 IoPPN, King’s College London, London, UK

**Keywords:** Cognition, Dementia, Memory, Attention, Neuropsychological, Alzheimers, Executive

## Abstract

**Background:**

The FDA have recently published draft guidance for the development of treatments for early Alzheimer’s disease. Key features of this guidance are the advocacy of sensitive cognitive measures and a taxonomy of disease severity. Whilst desirable patterns of cognitive-functional improvement are included, specific measures, and the magnitude of required effects, are not described.

**Main section:**

We describe key elements of the guidance content, especially with regard targeting key cognitive domains and the means by which they might be efficiently indexed in the disease stages included in the guidance. We discuss also the opportunities to assess cognitive performance in ‘Stage 2’ and ‘Stage 3’ patients, as well as the possibilities for effectively assessing function in the latter category. In this section we review candidate cognitive assessments that we judge are capable of delivering on the guidance specification for sensitive neuropsychological measures. This includes detailed consideration of the ADCS-PACC and Catch-Cog initiatives. With respect to the magnitude of effects, we propose that standardised effect sizes of 0.3 represent a reasonable level of efficacy based on the observation that already marketed drugs on average deliver this level of improvement.

**Conclusions:**

We propose the use of cognitive measures in stage 2 patients to index the cognitive skills known to be compromised early in the Alzheimer’s disease process. We recommend extending the traditional interest in episodic memory to include sensitive, reliable and valid measures of attention, working memory and aspects of executive function. We propose a focus on these additional cognitive abilities based on evidence that performance on tests of these domains is moderately well related to functional skills.

## Background

The FDA has recently published a new guidance note for developing drugs to treat early Alzheimer’s disease (AD) [1]. The guidance is as significant for what it doesn’t contain, as much as its actual content. For example, neither the ADAS-cog [2] nor the CDR-SB [3] are mentioned. One interpretation of this is that the agency recognises the risk that the mere mention of a test to illustrate a point will be seen as a specific endorsement of that measure. The absence of the CDR-SB will come as no surprise to those of us who attended the CTAD session at which an agency employee indicated that this would be the case. So much for what is absent, but what is actually in the document? The authors include guidance on the use of biomarkers and other aspects of drug development that will not be discussed here. However, cognitive assessment, and a four-stage taxonomy of disease progression are extensively discussed, and it is these topics that will be the focus of our consideration.

### General issues raised in the new guidance

The preamble to the guidance includes a helpful reminder that whilst we have tended to dichotomise cognition and function, the two are inextricably linked:

*‘FDA rejects this dichotomy and finds such usage inappropriate, because it implies that an effect on cognition itself, regardless of the nature of the observed effect and the manner in which it is assessed, cannot be clinically meaningful. This is certainly not the case’.*

A question this raises is how do we define what might be ‘clinically meaningful’? The guidance includes reference to cognitive changes ‘of a particular character, perhaps defined by magnitude or breadth of effect(s), may represent clinically meaningful benefit’. This issue has been a topic of discussion for some time and was considered by a number of us in a recent paper [4]. We will return to the topic of clinical meaningfulness later.

### Types of patients – a proposed taxonomy

Turning to the staging taxonomy, Stage 1 patients are in part defined as having no ‘detectable abnormalities on sensitive neuropsychological measures’. However, Stage 2 patients are defined as those who exhibit ‘detectable abnormalities on sensitive neuropsychological measures’ but in whom ‘there is no evidence of functional impairment’. The authors suggest in the context of Stage 2 patients that the ‘FDA will consider strongly justified arguments that a persuasive effect on sensitive measures of neuropsychological performance may provide adequate support for a marketing approval’. The authors suggest that a persuasive effect could be based on ‘A pattern of putatively beneficial effects demonstrated across multiple individual tests’.

Stage 3 patients are in part defined as exhibiting ‘subtle or more apparent detectable abnormalities on sensitive neuropsychological measures, and mild but detectable functional impairment’. The authors describe Stage 3 as ‘Early AD patients approaching the onset of overt dementia’ who are likely to have relatively mild but noticeable impairments in their daily functioning. The authors suggest that as with Stage 2 patients, ‘studies in this stage of disease will generally include sensitive measures of neuropsychological performance’. However, they recognise that this performance may be ‘of uncertain independent clinical meaningfulness’ and thus in their view ‘it is important to demonstrate that a drug favorably affects these functional deficits’.

### The assessment of Stage 2 Patients

So much for the guidance review, what might this mean in practice, and how might drug development sponsors respond? In the context of Stage 2 patients**,** in whom there are detectable cognitive but not functional deficits, we might sensibly employ a cognitive assessment that indexes the relevant domains of function. A number of such assessments have been developed and include computerised assessments, as well as traditional ‘paper-and-pencil’ (P&P) testing. Irrespective of the delivery platform the proposed assessments have tended to converge on the domains of episodic memory, working memory, and aspects of executive function. This focus has a long history and specific reference to these domains was included in the 2008 European Task Force guidance [5]. A very similar approach was adopted by the EPAD Scientific Advisory Group for Clinical and Cognitive Outcomes [6]. Specific examples of cognitive assessments that include measures of these domains are the Alzheimer Disease Cooperative Study Preclinical Alzheimer Cognitive Composite (ADCS-PACC) [7] and Catch-Cog [8] initiatives, both of which will be discussed in detail later in this paper. These assessments are composed of P&P measures, some of which have been combined with computerised assessments, as in the case of recently completed studies such as the one reported by Probiodrug [9], and in ongoing studies being conducted by companies such as Axon Neuroscience [10]. Whilst the content of these assessments has a similar conceptual basis, the specific composition of these assessments varies markedly. However, they converge with respect to exhibiting respectable levels of reliability, validity and sensitivity, and the domains of cognition they index. The guidance offered by Ferris et al. [11] and Harrison [12] provides the means for selecting appropriate measures. In the following section, we will describe current projects focused on developing novel and more comprehensive assessments of cognition. In the course of this description we will appraise these measures in the context of best practice guidance.

### ADCS-PACC

The ADCS-PACC is one of a number of composite cognitive measures employed in ongoing studies of early or preclinical AD [7]. It is a response to concerns that standard AD measures are likely to prove insensitive to early stage deficits and to cognitive change, whether as a function of disease progression and/or therapeutic intervention. The tests that comprise the ADCS-PACC have been selected to index primarily episodic memory, hence the inclusion of the Free and Cued Selective Reminding Test (FCSRT [13]) and the Logical Memory IIa sub-test from the Wechsler Memory Scale [14]. The former test yields a score of 0-48 and the latter from 0 to 25. Memory in the form of orientation is measured using the MMSE [15]. The fourth and final ADCS-PACC measure is the Digit Symbol Substitution Test (DSST) from the WAIS-R [16]. This has been selected by the authors as a measure of executive function, though the DSST is popularly regarded as measuring a number of cognitive functions, including both attention and working memory. The performance range for the DSST varies between 0 and 93.

The four components of the ADCS-PACC are all scored on different ranges and so in order to make performance on all four measures directly comparable a composite score of all ADCS-PACC tests is calculated using a z-score normalization method. Each of the four component change scores is divided by the baseline sample standard deviation of that component, to form standardized z-scores. These z-scores are summed to form the composite.

The tests that comprise the ADCS-PACC have a pedigree of use in various cross-sectional and longitudinal studies of patients with AD. The most notable of these is probably the ADNI group of studies. The cognitive focus of the assessment meets the requirement for measures of episodic memory, working memory and elements of executive function. The inclusion of the DSST also allows for praxis and attention to be assessed. The tests selected meet current best practice guidance and promise much as measures of longitudinal cognitive change. The ADCS-PACC has yet to demonstrate sensitivity to pharmacological treatment effects in studies of putative therapies for AD. However, the DSST has previously shown beneficial treatment effects in MCI [17]. A second key issue is whether the rates of cognitive change seen on these measures, when incorporated in assessments featuring multiple other tests, will be replicated when solely the ADCS-PACC measures are administered. This is a key element of validation and a question that must be asked of all new cognitive assessment tools, whether composed of entirely new tests, or measures that have been employed in previously conducted studies.

### Catch-Cog

A further approach is the possibility of employing measures that comprise traditional AD trial metrics, such as the ADAS-cog, NTB, etc. We have considerable experience with the use of these measures and an abundance of data with which to judge their sensitivity and reliability [18, 19]. A further advantage of this approach is that they are familiar to the drug development community, including trial sites, regulators, and other interested third parties. Repurposing well-known tests is therefore a tempting proposition. Catch-Cog is a composite score that blends memory components of the ADAS-cog with executive function measures from the NTB and ADCS-PACC [8]. Amongst the ADAS-cog subtests Word Recall, Word Recognition, and Orientation are substantially less prone to range restrictions than the other ADAS-cog measures (see Table 1) and have significant potential for capturing progressive decline in episodic memory and treatment effects. A point of note is that in contrast to the other ADAS-cog subtests, the Orientation test is naturally, if only partially, parallel in nature, and through the use of parallel word lists the Word Recall and Word Recognition stimuli can be varied across visits.

**Table 1:** Cognitive domains by instrument (# = Prone to ceiling (+) or floor (−) effects in mild stage patients)

Cognitive skill or domain	ADAS-cog subtest	ADCS-PACC	Catch-Cog
Episodic verbal memory test	Immediate Word Recall	FCSRT/MMSE	Immediate Word Recall
Confrontation naming	Naming Fingers and Objects # (+)	MMSE	–
Tests comprehension and praxis	Commands # (+)	MMSE	–
Episodic verbal memory test	Delayed Word Recall # (−)	Logical Memory/MMSE	–
Design copy	Constructional Praxis # (+)	MMSE	–
Familiar task execution	Ideational Praxis # (+)	MMSE	–
Semantic & episodic memory	Orientation	MMSE	Orientation
Episodic verbal memory test	Word Recognition	–	Word Recognition
Episodic memory	Remembering Test Instructions # (+)	–	–
Language production	Spoken Language Ability # (+)	MMSE	–
Semantic memory	Word-finding Difficulty # (+)	MMSE	–
Language comprehension	Language Comprehension # (+)	MMSE	–
Attention	Number Cancellation # (+)	DSST	DSST
Executive skills	Maze # (+)	DSST	DSST, COWAT, CFT, DS

An acknowledged deficiency of the ADAS-cog is the lack of quality measures of attention, working memory and executive function [20–22]. Valid and sensitive measures of these functions have been extensively employed in other composite measures [23]. For example, Catch-Cog includes the COWAT & CFT from the NTB, both of which are well-known and extensively validated tests of working memory and executive function [24]. These measures have also previously been shown to be sensitive to treatment effects, most obviously in the PBT2 [25] and Encenicline [26] studies. The final cognitive Catch-Cog measure is the Digit Symbol Substitution Test (DSST). As discussed above, this test has a number of virtues, including brevity (about two minute duration) as well as the capacity to measure a variety of cognitive skills, including attention and working memory. It is also acknowledged as a test of ‘timed executive function’ by the CHMP of the EMA [27]. As mentioned, this measure yielded a significant benefit of treatment with galanthamine in MCI [17] and has been observed to be a sensitive measure of treatment effects in other CNS indications [28].

The philosophy underlying selection of the Catch-Cog assessment is that robust, reliable, sensitive and valid measures will yield an efficient, evidence-based assessment of cognitive change. The selection of measures that index performance in clinically relevant skills, known to be compromised early in the Alzheimer’s disease process, meets expert group guidance on cognitive assessment. A further dimension of the Catch-Cog research program is that the Amsterdam Instrumental Activities of Daily Living [29] is being validated alongside the cognitive composite.

The measures selected for inclusion in Catch-Cog have a rich provenance for capturing treatment effects. The NTB executive function tests have previously performed well in studies of the PBT2 [30], as well as the Encenicline study mentioned above. A remaining but critical question for Catch-Cog is the extent to which it demonstrates robust psychometric characteristics, such as temporal reliability and the assessment’s capacity to show longitudinal change. Both issues are under active investigation. Given that the measures that comprise the Catch-Cog have independently exhibited robust psychometric attributes, acceptable levels of performance are anticipated in the validation study [31]. A summary of the cognitive domains and processes indexed by Catch-Cog, the ADCS-PACC, and ADAS-cog is shown in Table 1.

So, what would the FDA find ‘persuasive’ enough with respect to these measures to grant marketing approval for Stage 2 patient treatment? This is not specified, though the guidance authors suggest that this requires ‘A pattern of putatively beneficial effects demonstrated across multiple individual tests’ and that ‘A large magnitude of effect on sensitive measures of neuropsychological performance may also increase their persuasiveness’. The former requirement is the pattern of performance seen in recent studies of cognitive benefits of new multimodal anti-depressant drugs [32], captured using cognitive assessments the content of which is very similar to the above examples. The guidance authors do not provide an indication of how substantive a treatment would have to be. Currently marketed drugs yield positive cognitive effect sizes of about 0.3 [33] and we are necessarily left to speculate what the agency would regard as acceptable evidence. Intuitively we suspect that a standalone cognitive effect of a symptomatic relieving treatment might require positive effect sizes of at least 0.3 across a number of domains, with a 0.5 positive effect in a key area, such as episodic or working memory.

Now we turn to consider Stage 3 Patients, in whom the focus is on cognitive-functional impairment. Cognitive assessment is still ‘front and centre’ and the authors specify that ‘Ideally, the outcome measure used in this stage of disease will provide an assessment of meaningful cognitive function’. With respect to function, the authors point out that ‘Many of the assessment tools typically used to measure functional impairment in patients with overt dementia may not be suitable for use in these early stage patients’. They are presumably referring here to traditionally employed ADL assessments such as the DAD [34] and ADCS-ADL [35]. Consistent with past advice, the guidance authors allow for the use of ‘An integrated scale that adequately and meaningfully assesses both daily function and cognitive effects in early AD patients’ which would be ‘acceptable as a single primary efficacy outcome measure’. The inclination of sponsors with regard the selection of an integrated scale has been to employ the CDR-SB. This new guidance neither endorses nor prohibits the use of the CDR-SB. However, it is perhaps significant that the authors state that:

*‘FDA encourages the development of novel approaches to the integrated evaluation of subtle early AD (predementia) functional deficits/impact that arise from early cognitive impairment (e.g., facility with financial transactions, adequacy of social conversation)’.*

One supposes that this exhortation would be unnecessary if the CDR-SB was considered to be the sole acceptable solution to the need for a cognitive-functional measure. EMA has previously cautioned [36] that ‘The CDR-SB scoring requires extensive training and is subject to variability among ethnicity and languages’. It might be that current initiatives, such as Catch-Cog, in which we are seeking to validate the combined use of the more robust cognitive measures used in AD trials with the Amsterdam IADL will provide helpful alternatives to the use of the CDR-SB.

On a final topic, where does this new guidance leave us with respect to use of the ADAS-cog? A generous interpretation of the ADAS-cog’s utility is that it does a not bad job of measuring episodic memory, but that even after the addition of further measures [37] it is a wholly inadequate assessment of other important cognitive skills, such as attention, working memory, and aspects of executive function, especially in very early stage patients. Whilst it includes measures of language and praxis, they could in no sense be described as ‘sensitive neuropsychological measures’. Previously reported studies of very early stage patients have highlighted that performance on the majority of ADAS-cog subtests is at ceiling [17] and likely to stay there for the typical duration of most clinical trials featuring mild stage patients [38]. Abandoning the ADAS-cog for superior measures is long overdue and the quality of our clinical science will likely be enhanced by making the shift to more reliable, sensitive and valid measures.

## Conclusions

This new guidance offers welcome and helpful details of the FDA’s thinking with respect to the relationship between function and cognition, as well as the need for sensitive neuropsychological assessment. There is also helpful acknowledgement that the tools needed to determine efficacy will vary as a function of disease severity. Whilst still a draft document, it is encouraging to see the agency offering thoughtful guidance. This guidance appears to endorse the view that judicious cognitive domain targeting, wed to the selection of sensitive neuropsychological and cognitive measures, has the potential to demonstrate treatment efficacy in the very earliest detectable stages of AD.

## Abbreviations

AD: Alzheimer’s disease
ADAS-cog: Alzheimer’s disease Assessment Scale – cognitive subscale
ADCS ADL: Alzheimer’s disease Cooperative Study Activities of Daily Living
ADCS PACC: Alzheimer’s disease Cooperative Study Pre-Alzheimer’s Cognitive Composite
ADL: Activities of Daily Living
A-IADL: Amsterdam Instrumental Activities of Daily Living
CDR-SB: Clinical Dementia Rating scale – sum of boxes
CFT: Category Fluency Test
CHMP: Committee for Medicinal Products for Human Use
COWAT: Controlled Oral Word Association Test
CTAD: Clinical Trials in Alzheimer’s Disease
DAD: Disability Assessment for Dementia
DSST: Digit Symbol Substitution Test
EMA: European Medicines Agency
FCSRT: Free and Cued Selective Reminding Test
FDA: Food & Drug Administration
MCI: Mild Cognitive Impairment
MMSE: Mini-Mental State Examination
NTB: Neuropsychological Test Battery
WAIS-R: Wechsler Adult Intelligence Scale (Revised)
WMS: Wechsler Memory Scale
